# Child-to-Parent Violence as an Intervening Variable in the Relationship between Inter-Parental Violence Exposure and Dating Violence

**DOI:** 10.3390/ijerph17051514

**Published:** 2020-02-26

**Authors:** Izaskun Ibabe, Ainara Arnoso, Edurne Elgorriaga

**Affiliations:** Department of Social Psychology and Behavior Sciences Methodology, University College of Psychology, University of the Basque Country UPV/EHU, Avda. Tolosa 70, 20018-Donostia-San Sebastián, Spain; ainara.arnoso@ehu.eus (A.A.); edurne.elgorriaga@ehu.eus (E.E.)

**Keywords:** child-to-parent violence, dating violence, inter-parental violence, family violence, sexism

## Abstract

The exposure of adult children to inter-parental violence is an indirect form of victimization which has not been widely investigated in relation to its consequences in adulthood. The main goal of this study was to analyze predictors of dating violence based on an integrated model of intergenerational transmission of violence with the assessment of potential indirect effects of inter-parental violence exposure on dating violence through child-to-parent violence and sexism. A total of 847 college students participated in this study, ranging from 18 to 25 years of age. Inter-parental violence exposure plays a relevant role in dating violence, with indirect effects through child-to-parent violence and sexism. These results support social learning theory in explaining the intergenerational transmission of violence and indicate that further attention should be paid to children exposed to inter-parental violence. Intervention models to prevent the perpetration of dating violence should include the prevention of inter-parental violence exposure and child-to-parent violence.

## 1. Introduction

The main goal of this study was to assess the predictive model of dating violence based on an integrated model of intergenerational transmission of violence which includes inter-parental violence exposure, sexism, and child-to-parent violence (CPV). In this study, intimate partner violence (IPV) is defined as violent behavior between members of a couple either of an incidental nature or with a pattern of physical aggression, coercion, threats and/or control, regardless of gender. While IPV has traditionally focused on the marital context or on consolidated adult couples, dating violence has elicited growing interest in recent years [1]. When intimate partner violence is witnessed by children, the term used is inter-parental violence exposure. It might be helpful to distinguish between research on couples in committed, marital, or co-habituating relationships versus dating relationships. In dating relationships, members of the couple do not live together, are not economically independent from their parents, and do not have well-established relationships. Given these differences, there is a need for research focused on dating violence as separate from intimate partner violence.

### 1.1. Gender Symmetry Versus Directionality in IPV and Dating Violence

Although male-perpetrated IPV has long been a public concern, there is much empirical evidence to support that females are equally or even more likely to report perpetrating physical violence towards partners, particularly when referring to minor acts of aggression [2,3,4,5], college student dating violence [6], or adolescent dating violence [7]. Archer [2] concluded that in younger couples, gender differences were greater than in married couples, showing females perpetrating IPV more frequently than males.

Several studies have considered the expression of directionality of violence perpetrated toward a partner in heterosexual couples, distinguishing between unidirectional and bidirectional violence [8,9]. There are three mutually exclusive categories, or dyadic concordance types [9]: Unidirectional male-to-female, unidirectional female-to-male, and bidirectional. Unidirectional violence occurs when only one member of the couple is a perpetrator and the other is a victim [8]. Thus, a member of an intimate partner relationship could be a victim, a perpetrator, or victim and perpetrator at the same time. With respect to the prevalence of IPV, bidirectional violence has been found to be the largest category by far, with the least frequently occurring being unidirectional male-to-female violence [5].

In the review conducted by Langhinrichsen-Rohling et al. [8], which includes physical and psychological IPV, bidirectional violence was the most common IPV pattern in all types of samples (e.g., large population samples, small community samples, students, clinical samples, or legal/criminal samples). However, the ratio of unidirectional female-to-male violence was slightly higher than that of unidirectional male-to-female in large population, smaller community, and school studies, while the unidirectional male-to-female ratio was higher in criminal justice studies. Moreover, these authors indicated that bidirectional violence is not always symmetrical. Sometimes, perpetration of violence is likely to be initiated out of self-defense, and it is possible that the violence of the two partners is not matched in severity and frequency.

There are few studies regarding adult children’s exposure to inter-parental violence. One study by Straus and Michel-Smith [10] based on 15 nations found that overall about 14% of students reported one or more instances of physical assault between their parents during the year they were 10 years old, and 6% reported a severe assault. They reported unidirectional father-to-mother (25%), unidirectional mother-to-father (22%), and bidirectional violence (53%). Additionally, they indicated that inter-parental violence exposure was a chronic pattern in about three quarters of cases. Thus, it would be important to study the harmful effects of exposure to inter-parental violence of children both in childhood and adulthood.

### 1.2. IPV, Dating Violence and Sexism

There is a belief that patriarchal values lead to potentially violent behavior when couples are in situations of conflict or disagreement due to an unequal balance of power between the male and female partners. Sexism is a form of complex prejudice against women which has become increasingly scientifically relevant over the last decades. The theory of ambivalent sexism proposes two opposite evaluative orientations toward women: Hostile sexism (traditional sexism based on prejudice against women) and benevolent sexism (stereotypical paternalistic attitudes and overprotection towards women, which are subjectively positive) [11]. However, both types of sexism are used to justify men’s structural power and to maintain gender inequalities because they restrict women to a lower social status.

Males in a community population showed more sexist beliefs than females [12], with similar results in a college student population [6,13]. The expected strength of the relationship between sexist beliefs and IPV or dating violence would be moderate according to previous studies. In a meta-analysis composed of 85 studies based on adult populations, including both types of study of IPV and gender violence, authors concluded that traditional gender attitudes and ideologies in men have a moderate effect size (*r* = 0.29) in relation to the perpetration of physical violence against their partner [14]. Additionally, they found that in the adult population, hostile or accepting attitudes of violence (e.g., patriarchal domination) against women in intimate partner relationships predicted the violence exerted by both men and women moderately or weakly [15,16]. Similarly, the results of studies based on dating violence are comparable in college student populations [6,13]. In this context it would be interesting to explore the potential relationship between father-to-mother violence exposure and sexism, because children could learn certain prejudices against women through witnessing violent or disrespectful behavior by their father toward their mother.

### 1.3. Inter-Parental Violence Exposure, CPV and Dating Violence

The exposure to inter-parental violence of children has been associated with more strongly aggressive behavior by them in several contexts. There is a great deal of literature about the bidirectionality of violence hypothesis in the context of CPV [17,18,19,20,21,22]: Children who have experienced parent abuse or have observed inter-parental violence tend to be more violent toward parents. In a community population, child aggression could represent a functional response to family strain or an attempt to cope with inadequate parenting education [17].

CPV has received growing interest during the last decade due to the increase of complaints filed by parents, according to the General Prosecutor’s Office of Spain [23]. This criminal modality remains consolidated as an evil endemic to society, and the Juvenile Court indicates that these crimes are among those presenting the greatest difficulties. However, the number of cases in which parents do not report their children’s behavior to the Juvenile Court remains unknown. In their review of community sample prevalence data, Simmons, McEwan, Purcell, and Ogloff [24] estimated the 12-month incidence of adolescent-perpetrated physical CPV to be between 5% and 21%. Although CPV perpetration is particularly related to age, it does not end at the age of 18; in the US, it has been found that 10% of assaults committed by young adults (e.g., 18–25 years old) were directed at parents [25]. In Spain, the study by Gámez-Guadix, Jaureguizar, Almendros, and Carrobles [26] examined the prevalence of CPV in a sample of 1343 university students, and the prevalence of physical aggression against parents was around 5%. These data reflect the magnitude of this social problem.

The current definition of CPV includes different forms of abuse (physical, psychological, and financial), the perpetrator’s awareness of the violent behavior and repeated perpetration, excluding isolated acts of violence [27]. On the one hand, CPV research has operationalized the term “child” for perpetrators younger than 18 years, but data from different countries reveal that at least half the young adults between 18 and 24 continue living with their parents, with little research extending beyond the age of 18 [24]. On the other hand, although early childhood aggression is an important topic for study, it should be excluded from the conceptualization of CPV because very young children do not possess the capacity to act in an abusive way. Thus, it would be interesting to take into account the age of children when considering CPV and include adolescents and young adults living with parents. This is an important gap in the literature on CPV, and one relevant to the current study.

Although mixed results have been found with respect to gender differences in the perpetration of physical CPV, Ibabe and Bentler [28] suggest that gender differences in this type of violence depend on its seriousness. In cases of serious violence, males are more likely to be aggressors, whereas in situations of mild violence there are no differences, or they are less likely to engage in it. However, it seems that in community populations at least, psychological aggression is more frequent in girls than in boys [28,29,30]. In judicial contexts, both types of aggressive behavior are usually reported together [30]. Moreover, some personality traits have been found in young people who are aggressive toward their parents, such as traits of antisocial personality [29], as well as depressive symptoms, substance use [24,31], and aggressive family discipline [25,32]. The relationship between substance use and CPV possibly reflects the recognized relationship between substance use and general aggression in community [24].

It is possible that CPV by adolescents or young adults is an antecedent of dating violence perpetration in heterosexual couples, but studies of this are still scarce [33], and it would be timely to analyze underlying mechanisms that explain this association. Such a link would imply that CPV might play a mediational role between inter-parental violence exposure and dating violence. Moreover, it was found that juvenile offenders for CPV use more direct and indirect forms of violence in their relationships with their peers [34]. Thus, several authors have attempted to explain CPV based on single-theory theoretical frameworks (social learning, feminist, evolutionary, and family systems theories) and a multifactor framework (ecological model) [24]. Social learning theory has been applied extensively to the understanding of aggression [35]. Social learning would imply that children learn forms of aggression (physical and psychological) similar to those they have witnessed in inter-parental relationships. Thus, children could internalize that physical and verbal coercion are adequate and acceptable strategies to change someone else’s behavior, and children could express different externalizing symptoms as CPV [19]. Children learning to solve their conflicts using violent behavior may manifest violent behavior towards parents when they reach adolescence, and they could show violence in their dating relationships. The inter-generational transmission of violence could be explained through social learning processes in the context of family socialization. Nevertheless, a meta-analysis by Stith et al. [36] reported a weak-to-moderate relationship between inter-parental violence exposure and later dating violence. Along similar lines, Calvete, Fernández-González, Orue, and Little [37] found evidence of a small effect of inter-generational transmission of family violence in adolescents, indicating that cognitive and emotional schemas act as mechanisms for dating violence. However, Kinsfogel and Grych [38] indicated that exposure to inter-parental conflict was associated with a greater use of physical and verbal aggression in dating relationships among boys but not girls. Furthermore, associations are stronger when IPV is conceptualized broadly (physical, psychological, and/or sexual aggression) rather than narrowly (physical violence only). According to the rejection sensitivity model, it was found that exposure to family violence at home was related to the hostile social perception of adolescents in CPV cases [18]. Repeated hostile behavior by parents may increase the risk of later violent behavior both in family-related (CPV) and in dating-related contexts (dating violence).

Omer [39], who developed the non-violent resistance approach for behavior problems of children, indicated that abusive adults need not necessarily have been abused in their childhood or exposed to inter-parental violence, but may have perpetrated violence as a child against their parents. In order to turn into abusive adults, children must have the opportunity to perpetrate violent behavior. Indeed, some studies have shown that the best predictor of adult violence is the violence against parents or siblings perpetrated when they were children, rather than that suffered as the intended recipient [40]. As there is limited empirical research regarding CPV in early adulthood [24], it would be necessary to investigate this issue. Understanding the intervening variables that might help identify who is at risk of becoming involved in dating violence during young adulthood is relevant for decreasing the prevalence of IPV.

### 1.4. Objectives and Hypotheses

One objective of the current research was to provide basic descriptive information about the prevalence rates of different types of inter-personal violence (children’s exposure to inter-parental violence, CPV, and dating violence) in early adulthood and the variations involved (directionality and the severity of violence which characterizes each of the types).

However, the main goal of the study is to test a theoretically derived process of inter-parental violence transmission, including in the model inter-parental violence exposure, CPV, and ambivalent sexism. Two potential intervening factors (CPV and ambivalent sexism) were assessed to explain the relationship between inter-parental violence exposure and dating violence. The focus on the link between parents’ violence toward each other and their young adult children’s violence toward their parents is novel. Intergenerational transmission of violence is best evaluated by assessing parents’ violence toward each other and young people’s violence toward their parents by looking at what happened to college students in the previous year. Inter-parental violence could have begun much earlier in life, when the college students were children.

The hypotheses of this study are:

As a study based on a college student sample, we predict that the rate of unidirectional father-to-mother physical violence would be equal to or slightly higher than unidirectional mother-to-father physical violence. There is some evidence in the literature to support this hypothesis when college students report on inter-parental violence [10].

In heterosexual relationships, the prevalence rate of physical dating violence perpetration will be between 3.8% and 41.9% due to the great variability found in previous studies [41,42], and females will perpetrate more physical dating violence than males when technical abuse criteria are applied [4,5].

The prevalence rate of physical CPV will be between 5% and 21% [24], and females will present more psychological CPV than males with technical abuse criteria [29,30,31].

CPV will mediate the relationship between inter-parental violence and dating violence. Previous studies have shown inter-parental violence exposure to be a relevant risk factor for CPV [19,43], and perpetration of CPV would be expected to be associated positively with dating violence, based on a longitudinal study [33]. Thus, family environment could be considered to be the first social laboratory in which children internalize patterns of conflict resolution. For this reason, CPV could also be considered a previous stage for dating violence, because the young people involved have gained experience as perpetrators of violence toward family members.

It is hypothesized that ambivalent sexism would have a mediating effect in the relationship between inter-parental violence exposure and dating violence. Although there are no previous studies regarding the association between inter-parental violence exposure and ambivalent sexism, it makes sense to think that children could develop certain sexist beliefs or prejudices against women through the witnessing of violent or disrespectful behavior of fathers toward mothers. Ambivalent sexism will be a moderate predictor of dating violence, taking into account two studies based on Spanish college students [6,13].

## 2. Materials and Methods

### 2.1. Participants

This study involved 1209 college students (67% of whom were women) from the Basque Country (Spain), with an average age of 19.65 years (ranging from 18 to 25 years) and a standard deviation of 1.75 years. However, the number of participants included in all relevant analyses was 847 after excluding students who had not had any heterosexual dating relationships. Selection of the full sample of participants was performed using a non-random sampling method on eight university degree courses at the University of the Basque Country. We sought to obtain a representative sample of the population taking into account gender, university degrees, and language (Spanish or Basque). There were different class groups depending on language in each university college. All students of this university can choose the language that they study in for each academic year. Of these participants, 53% were enrolled in degrees in the social sciences, 33% in health sciences, and 14% in scientific and technical fields. Participants living with their family made up 67% of the sample, with 30% in student housing and with their family on non-college days, and 3% were independent from their family. The participation rate for 18-to-25-year-olds from families with a monthly net income of between €1,000 and €3,000 was notably higher (73%) than for lower income participants (10%), and the participation pattern did not vary too much once family income exceeded €3,000 (17%). In the Basque Country in 2014, the year in which the data were collected, average monthly net income was around €2,000.

### 2.2. Variables and Instruments

In order to standardize the answer format in all instruments, a five-point Likert scale was used. The frequency of violent behavior was measured on the following scale: 1 = Never, 2 = Rarely, 3 = Sometimes, 4 = Often, 5 = Very often. It is important to specify the timing of the different types of violence assessed. Participants were asked to take the previous year of living together as a reference to answer about the exposure to inter-parental violence and CPV. Dating violence was based on the current partner or the last dating relationship in the past year.

**Inter-parental violence exposure** Revised Tactics Scales, CTS2; [44]; Spanish version [45]. An adaptation of the two subscales (physical assault and psychological aggression) was made to assess the exposure to violence between parents, with 10 items in total. These items were bidirectional (father-to-mother vs. mother-to-father), and there were 5 items in each direction. Physical violence (4 items) (e.g., my father has pushed or hit my mother in a fight) and psycho-emotional aggression (6 items) (e.g., my father shouted at my mother) in the previous year of parents and children living together were assessed. In this study, alpha coefficients were excellent for inter-parental physical father-to-mother violence (α = 0.89) and physical mother-to-father violence (α = 0.83), while for global violence father-to-mother (α = 0.75) and mother-to-father (α = 0.77), they were acceptable.

**Child-to-parent violence** [46]. Participants responded to six parallel (father/mother) questions from this scale (items from CTSPC Conflict Tactics Scales, Parent-Child Version) to measure the occurrence of physical and psychological CPV in past year of living together. Physical CPV was assessed with three items (e.g., I slapped or punched my father/mother); and other three items assessed verbal CPV (e.g., I shouted at my mother/father). For this sample, the internal consistency for physical violence (α = 0.57) and psychological aggression (α = 0.81) were tolerable.

**Victimization of dating violence** (Dating Violence Questionnaire, Cuestionario de Violencia entre Novios, CUVINO, [47]). This instrument is composed of 42 behavioral indicators. Answers are based on the current partner or last partner in the last year of relationship. These indicators are grouped into eight dimensions of violence: Physical, sexual, coercion, gender, emotional punishment, detachment (e.g., has ignored your feelings), humiliation (e.g., humiliates you in public), and instrumental (e.g., has stolen from you). The overall internal consistency in this study (α = 0.92) was excellent. In this study, the overall internal consistency (α = 0.92) was excellent, and the eight dimensions had quite acceptable internal consistency: Physical (α = 0.74), sexual (α = 0.79), coercion (α = 0.77), gender (α = 0.73), emotional punishment (α = 0.66), detachment (α = 0.81), humiliation (α = 0.79), and instrumental (α = 0.65). The information about heterosexual partners was based on two items: (a) Respondent’s sex; male or female, and (b) respondent’s last partner; male or female.

**Perpetration of dating violence** (Perpetration of Dating Violence Scale). A questionnaire of six items was drawn up ad hoc in order to assess perpetration of dating violence, without repeating the items used in CUVINO [47] to avoid repetition bias. Answers were based on the current partner or last partner in the last year of relationship. This scale was composed of three items on physical violence in dating (e.g., when my partner and I get angry, we are usually abusive and physically assault each other) and three more on psychological aggression (e.g., I usually initiate an argument by yelling, insulting, or threatening my partner). In a principal axis factoring analysis with oblimin rotation, the total explained variance of two factors was 43.75%. The first factor (eigenvalue = 1.56) combined items related to psychological aggression, and the second factor (eigenvalue = 1.05) items related to physical violence. All items had factor loadings above 0.50. The predictive validity of this scale is supported by the association of dating violence perpetration with dating violence victimization (*r* = 0.50, *p* < 0.01), father-to-mother violence exposure (*r* = 0.19, *p* < 0.01), and mother-to-father violence exposure (*r* = 0.17, *p* < 0.01). In this study, the overall internal consistency of this scale (α = 0.72) was acceptable, but the psychological aggression subscale (α = 0.69) and the physical violence subscale (α = 0.59) did not reach the desirable level (α ≥ 0.70). This could be due to the relatively small number of items in each of these subscales.

**Ambivalent sexism** (Ambivalent Sexism Inventory, ASI, [11]; Spanish version [48]. This scale is composed of 22 items with 5 response options (1 = strongly disagree; 5 = strongly agree) that measure hostile sexism (e.g., women exaggerate the problems that they have at work) and benevolent sexism (e.g., women should be cherished and protected by men). Internal consistency of hostile sexism (α = 0.90), benevolent sexism (α = 0.86), and ambivalent sexism (overall) (α = 0.92) were excellent.

**Demographics**. A single item assessed sex (1 = female, 0 = male), age in years was a continuous variable, and type of college degree, as well as educational level of parents and family income were included.

### 2.3. Procedure

Approval for this research was issued by the Ethics Committee of the University of the Basque Country and the ethical code number is CEISH/25112014. While sample selection of participants was performed using a non-random sampling method at the University of the Basque Country, we nevertheless tried to obtain a representative sample of the population taking into account sex, university year, and degree. The instructions for data collection were standardized and described in a step-by-step manner. Data collection took place during the 2014–2015 academic year in the presence of staff assigned to this research and took approximately 45 min. Once the data were analyzed, a report containing the general findings was issued to the corresponding lecturers to inform the students of their respective class groups.

### 2.4. Data Analysis

First, the prevalence rates of inter-parental violence exposure, dating violence, and CPV were calculated applying zero tolerance criteria (when the response “rarely” or more in terms of frequency was given in response to any item) and technical abuse criteria (when the response “sometimes” or more in terms of frequency was given in response to any item) [49]. Prevalence rates of physical violence and psychological aggression were calculated for inter-parental violence exposure, dating violence, and CPV, as well as directionality of inter-parental violence exposure. Participants answered with regard to their current relationship or last intimate relationship. In order to analyze sex differences of the three types of violence, the Chi-square test was used.

From a victimization perspective, psychological aggression was calculated including six dimensions of CUVINO (detachment, humiliation, coercion, gender, emotional punishment, and instrumental). As the perpetration and victimization of dating violence were measured with different instruments, the directionality of violence was not analyzed. Prevalence analyses of dating violence and the predictive model included only participants who had had heterosexual dating relationships (*N* = 847). A correlation matrix was calculated (see Table 1), in which 9 observed variables were included with their means and standard deviations.

The EQS 6.2 software, Structural Equation Program (Multivariate Software Inc., Encino, CA) was used to assess whether the proposed models were adequate. An initial confirmatory factor analysis (CFA) assessed the adequacy of the hypothesized measurement model and the associations between sex as an observed variable and the latent variables and female: Inter-parental violence exposure (indicators: Physical violence father-to-mother, physical violence mother-to-father), ambivalent sexism (indicators: Hostile, benevolent), CPV (physical, psychological), and dating violence (indicators: victimization, perpetration). Then, a structural model posited female sex as a predictor of dating violence perpetration, psychological CPV, and the ambivalent sexism factor. Dating violence was predicted by ambivalent sexism, inter-parental Violence Exposure and CPV. In turn, Inter-parental Violence Exposure predicted CPV and ambivalent Sexism, and mediational variables of CPV and sexism were tested. The estimation of indirect effects was accomplished using a SEM model because mediational variables are latent factors. In order to interpret indirect effects, asymmetrical confidence intervals were followed, as recommended by MacKinnon, Lockwood, Hoffman, West, and Sheets [50], using Mplus 7.4. MacKinnon et al. [50] proposed constructing asymmetric confidence limits using bias-corrected bootstrap sampling methods to accommodate the non-normal distribution of the intervening variable effect based on the distribution of the product of random variables. If the confidence interval does not include zero, the intervening variable effect is significant.

In addition, three alternative SEM models were assessed. The first alternative model assessed the impact of dating violence on CPV (the opposite path to that indicated in the original model), holding all other paths constant. This model was planned to test the degree to which CPV by young adults is an antecedent of dating violence perpetration. In the second alternative model, a change in the indicators of the latent inter-parental violence exposure factor was made from the original model. Specifically, physical violence and psychological aggression were included in each of the two observed variables (father-to-mother violence and mother-to-father violence) instead of only inter-parental physical violence. This model was tested because in a college student population, witnessing physical parental violence will probably be quite low. In the third alternative model, the original SEM model with living status (1 = with parents; 0 = other situation) as a control variable was tested. It would be interesting to test if inter-parental violence exposure is different as a function of participants living with their parents or not.

The goodness-of-fit of the models was assessed with the normal theory maximum-likelihood (ML) chi-square, the Satorra and Bentler [51] robust maximum-likelihood (S-B) chi-square statistics, the comparative fit index (CFI), and the Satorra-Bentler robust comparative fit index (RCFI). Robust statistics are more appropriate when the data are not multivariate normal (Mardia’s normalized coefficient exceeded 174.14). Non-normality was substantially driven by variables related to violence, which, as expected, had highly non-normal marginal distributions: Inter-parental violence mother-to-father (skewness = 6.84, kurtosis = 57.74), inter-parental violence father-to-mother (skewness = 6.33, kurtosis = 49.18), child-to-parent physical violence (skewness = 2.86, kurtosis = 9.14), dating violence perpetration (skewness = 3.06, kurtosis = 12.88), dating violence victimization (skewness = 3.14, kurtosis = 15.04). A value equal to or greater than 0.95 was desirable for the CFI and RCFI. The root mean square error of approximation (RMSEA) was also used to assess goodness of fit, with a value of 0.06 or less being desirable [52]. Fit indices based on normal theory and the robust statistics are reported, while *p*-values of regression coefficients are based on robust statistics. Moreover, for indirect effects bootstrapped symmetrical confidence intervals and *p*-values are included.

## 3. Results

### 3.1. Prevalence Rates of Different Types of Violence

Inter-parental violence taking into account the direction of violence. On the one hand, the prevalence rate of physical inter-parental violence exposure in accordance with zero tolerance criteria was 9.7% in the previous year of living together (unidirectional father-to-mother 3.3%, unidirectional mother-to-father 3.5%, and bidirectional violence 2.9%), while with technical abuse criteria the prevalence rate reported was around 3.2% (father-to-mother 1.4%, mother-to-father 1.1%, and bidirectional violence 0.7%). There were no statistical differences between unidirectional father-to-mother violence and mother-to-father violence with tolerance criteria, χ^2^(1, *N* = 78) = 0.05, *p* = 0.821, or with technical abuse criteria, χ^2^(1, *N* = 30) = 0.53, *p* = 0.465. On the other hand, the prevalence rate of psychological inter-parental aggression exposure in accordance with zero tolerance criteria was 83.1% in the past year of living together (unidirectional father-to-mother 1.9%, unidirectional mother-to-father 11.2%, and bidirectional violence 70%), while with technical abuse criteria, the prevalence rate reported was 50.7% (father-to-mother 5.3%, mother-to-father 14.5%, and bidirectional violence 30.9%). Unidirectional mother-to-father psychological aggression was significantly higher than father-to-mother violence with zero tolerance criteria, χ^2^(1, *N* = 126) = 34.57, *p* < 0.001, and with technical abuse criteria, χ^2^(1, *N* = 430) = 268.83, *p* < 0.001.

Prevalence rates of CPV and dating violence. The perpetration rates of physical CPV (14% zero tolerance; 5% technical abuse) was much lower than psychological CPV (94% zero tolerance; 67% technical abuse). Moreover, perpetration of physical dating violence in heterosexual couples was 10.3% in accordance with zero tolerance criteria, while around 3.5% of participants showed technical abuse. Based on a victimization perspective, prevalence rates were similar between the zero tolerance criteria (10.4%) and technical abuse criteria (2.7%). Perpetration rates of psychological dating aggression were lower (40% zero tolerance; 10% technical abuse) than victimization rates (75% zero tolerance; 40.7% technical abuse).

With respect to sex differences, females (49%) showed a higher prevalence rate of psychological CPV than males (18%) using technical abuse criteria, χ^2^(*N* = 1,202) = 35.36, *p* < 0.001. However, there were no sex differences for physical CPV, χ^2^(*N* = 1,179) = 0.076, *p* = 0.783. When sex differences in heterosexual dating violence were analyzed using technical abuse criteria, females (3.1%) perpetrated more physical violence than males (0.4%), χ^2^(*N* = 847) = 5.08, *p* = 0.024, as well as more psychological aggression (females 9.7% and males 3%), χ^2^(*N* = 827) = 10.27, *p* < 0.001.

### 3.2. Correlation Matrix among Observed Variables

To examine the relationships among observed variables, a correlation matrix was computed (see Table 1). It is noticeable how mother-to-father violence was related to CPV (physical *r* = 0.237; *p* < 0.01; psychological *r* = 0.151; *p* < 0.001), but father-to-mother violence was related to dating violence perpetration (*r* = 0.189, *p* < 0.01), dating violence victimization (*r* = 0.138, *p* < 0.01), and benevolent sexism (*r* = 0.105, *p* < 0.01). Moreover, female sex was associated with more psychological CPV (*r* = 0.085, *p* < 0.05) and dating violence perpetration (*r* = 0.108, *p* < 0.01).

### 3.3. Confirmatory Factor Analysis

In the confirmatory factor analysis, all variables had factor loadings higher than 0.50, and they were significant (*p* < 0.001). Fit indexes for the CFA model which required no model modification were all excellent: ML χ^2^ (18, *N* = 847) = 55.61, *p* < 0.001; CFI = 0.962, RMSEA = 0.056; S-B χ^2^ (18, *N* = 847) = 46.59, *p* < 0.001; CFI = 0.944, RMSEA = 0.049.

### 3.4. Structural Path Model

**Figure 1:**
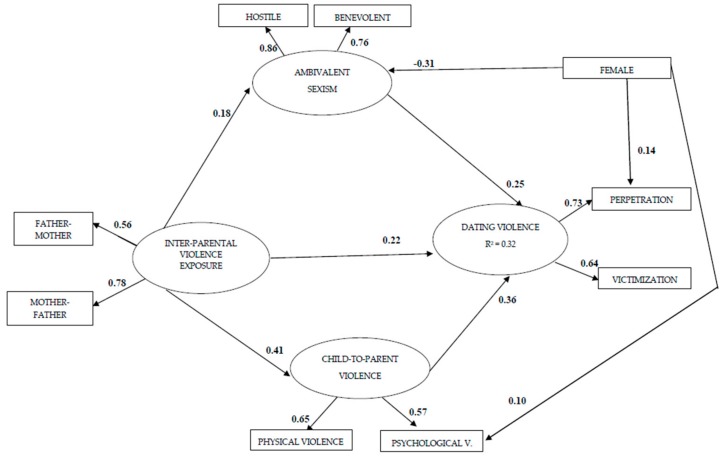
Structural model predicting dating violence based on 847 participants from a university student population. All estimated parameters are standardized. All factor loadings and regression coefficients are significant at *p* < 0.05. The SEM model included participants who had had heterosexual dating relationship.

The predictive structural model of dating violence is presented in Figure 1. The structural model had excellent fit statistics based on normal theory: ML χ^2^ (20, *N* = 847) = 54.27, *p* < 0.001; CFI = 0.965, RMSEA = 0.051. In general, fit indicators were better with the robust method: S-B χ^2^ (20, *N* = 847) = 44.57, *p* < 0.01; CFI = 0.953, RMSEA = 0.043. This model accounted for 32% of the variance in dating violence. Inter-parental violence exposure presented direct effects on CPV (*β* = 0.41, *p* < 0.001), and dating violence (*β* = 0.22, *p* < 0.01). At the same time, CPV (*β* = 0.36, *p* < 0.001) and ambivalent sexism (*β* = 0.25, *p* < 0.01) showed positive direct effects on dating violence. Moreover, inter-parental violence exposure significantly predicted ambivalent sexism (*β* = 0.18, *p* < 0.01). Additionally, female sex was associated with less ambivalent sexism (*β* = −0.31, *p* < 0.001), more dating violence perpetration (*β* = 0.14, *p* < 0.001), and more psychological CPV (*β* = 0.10, *p* < 0.05).

The first alternative model sought to analyze the impact of dating violence on CPV and fits the data reasonably well overall, with similar results to the model presented in Figure 1, ML χ^2^ (20, *N* = 847) = 49.49, *p* < 0.001; CFI = 0.970, RMSEA = 0.047; S-B χ^2^ (20, *N* = 847) = 40.34, *p* < 0.05, CFI = 0.961, RMSEA = 0.039. Dating violence was a significant predictor of CPV, and the regression coefficient was similar to the original model (*β* = 0.40, *p* < 0.001). The second alternative model, which included indicators of inter-parental violence exposure the global father-to-mother and mother-to-father violence, also fits the data well, ML χ^2^ (20, *N* = 847) = 63.98; *p* < 0.001; CFI = 0.965, RMSEA = 0.058; S-B χ^2^ (20, *N* = 847) = 49.20, *p* < 0.05; CFI = 0.960; RMSEA = 0.047, but explained variance in dating violence (R^2^ = 0.19) was 40% lower.

In the third alternative model, the original SEM model with living status as control variable was tested, but living status (with parents or other situation) was not related to inter-parental violence exposure (*β* = −0.05, *p* > 0.05), CPV (*β* = −0.09, *p* > 0.05) nor dating violence (*β* = -0.08, *p* > 0.05), ML χ^2^ (26, *N* = 847) = 80.39; *p* < 0.001; CFI = 0.945, RMSEA = 0.056; S-B χ^2^ (27, *N* = 847) = 68.28, *p* < 0.001; CFI = 0.927; RMSEA = 0.049. This model accounted for 19% of the variance in dating violence.

Moreover, indirect effects mediated through the intermediate variables were examined. Inter-parental violence exposure had a significant positive indirect effect on dating violence, mediated through CPV (*β* = 0.15, *p* < 0.01) and mediated through sexism (*β* = 0.03, *p* < 0.05). In order to test if these indirect effects were significant, bootstrapping was used to calculate confidence intervals and *p*-values. An examination of the specific indirect effects indicates that CPV is a mediator, as its 95% CI does not contain zero (0.048, 0.301) and this specific effect is significant (*p* = 0.003), which is also the case with ambivalent sexism (with 95% *CI* 0.009, 0.081 and *p* = 0.015). These results indicate that the mediational effect of CPV in the relationship between inter-parental violence and dating violence is significant and low-medium, while the mediational effect of ambivalent sexism is significant but small.

## 4. Discussion

The main objective was to analyze an integrated model of intergenerational transmission of IPV, including inter-parental violence exposure, CPV, and ambivalent sexism. This model included two intervening factors (CPV and ambivalent sexism) to explain the relationship between inter-parental violence exposure and dating violence. Strong empirical evidence was found in favor of the proposed predictive model of dating violence based on a college student population. This study also aimed to study the directionality of young adult children’s exposure to inter-parental violence in early adulthood, and prevalence rates of CPV and dating violence taking into account sex differences.

### 4.1. Directionality of Inter-Parental Violence Exposure

The first hypothesis predicted that the rate of unidirectional father-to-mother physical violence would be equal to or slightly higher than unidirectional mother-to-father physical violence. Exposure of young adult children to physical inter-parental violence during the previous year of living together was 9.7% or 3.2%, according to the zero-tolerance or technical-abuse approach. Straus and Michel-Smith [10] found that college students in 15 nations reported rates of 14% physical violence between their parents and 6% severe assault when they were 10 years old. As predicted, the rate of unidirectional mother-to-father physical violence (3.5%) was equal to unidirectional father-to-mother physical violence (3.3%). This result is consistent with the findings of Straus and Michel-Smith [10], based on a student college sample and gender symmetry studies which report similar rates of physical aggression perpetration towards their partner [2,3,4,5]. In order to develop effective interventions, it is necessary to know the nature of IPV (subtype of unidirectional or bidirectional violence), and this requires knowledge of the context in which the violence occurs or motivations for violence perpetration. Different intervention models may be required in cases where perpetrators are also victims of IPV, when males are exclusively the perpetrators or exclusively victims [53].

### 4.2. Prevalence of Dating Violence and Sex Differences

The hypothesis regarding the physical dating violence perpetration rate was confirmed. In the current study, perpetration of physical dating violence in heterosexual couples was approximately 10% for any physical aggression (zero tolerance criteria) and 3% for physically abusive behavior (technical abuse criteria), based on both perpetration and victimization perspectives. The rate of physical dating violence perpetration was expected to be between 3.8% and 41.9% due to the great variability found in previous studies [42]. In a Spanish study of all the forms of slight physical aggression analyzed in 1886 university students, about 15% of youths surveyed admitted having restrained, hit, or kicked and/or shoved their partner during the current or past relationships [54]. In a study based on teen dating violence with Spanish sample differentiated between occasional violence (14%) and frequent violence (7%) [41], and prevalence rates tend to be slightly higher.

Moreover, as expected, gender differences were found regarding dating violence. According to previous studies based on college student samples [5], females perpetrated slightly more physical dating violence using technical abuse criteria in heterosexual relationships than males, but the size effect was small (*r* = 0.08). Recently, some authors have pointed out the need to investigate measurement equivalence as a standard procedure for all instruments measuring intimate partner abuse when comparisons across males and females are made [55]. This would mean that it is likely biased to compare mean levels of perpetration between men and women when behavior-based rating scales are used. Thus, future research should explore measurement equivalence across sexes in the reporting of dating violence.

### 4.3. Prevalence of CPV and Sex Differences

Our hypothesis predicted that the prevalence rate of physical CPV would be between 5% and 21% [24]. In the current study, physical CPV of young people during the past year of living together was 5%, according to the technical-abuse approach and 14% with zero-tolerance. This prevalence rate is consistent with past research with adolescents [21] and with young adults [56]. Ibabe et al. [20] found that 5% of teenagers had exercised some form of severe physical violence towards their parents at some time during the previous year. In a study of young Australians aged 14–25 years, Simmons et al. [56] reported a similar prevalence rate of physically abusive behavior (7%) toward one parent. This means that future research should consider CPV in those above the age of 18 because many young people still live with their parents after becoming legal adults, although it would not be necessary for parents and children to reside together for abuse to happen. Risk factors and patterns of abuse may evolve with age [24].

As expected, daughters were more frequent perpetrators of psychological CPV than sons using technical abuse criteria. This result is consistent with the findings of a recent study by Rico et al. [30], in which a sample composed of 934 students aged between 13 and 21 years was analyzed, and daughters perpetrated more psychological CPV in comparison with sons.

### 4.4. Mediational Effect of CPV

The fourth hypothesis indicated that inter-parental violence exposure of children would moderately predict dating violence, with mediating effects of CPV. The structural model of dating violence, including victimization and perpetration, explained 32% of variance. The data are consistent with the proposed theoretical model in the current research, which includes CPV and sexism as intervening variables in the relationship between inter-parental violence exposure and dating violence. The hypothesis of bidirectionality of family violence is well known [18,19,20] and indicates that children who have observed inter-parental violence or suffered parent-to-child abuse tend to be more violent toward parents. Thus, it is not surprising that young adults between 18 and 25 years of age who witness inter-parental violence are also more likely to direct violent behavior toward their parents.

The perpetration of CPV was a valid predictor of dating violence perpetration and victimization among young men and women. With the exception of a study by Izaguirre and Calvete [33], there are few studies that have analyzed the relationship between these two variables with adolescents. The results indicate that adolescents who act aggressively toward parents tend to act aggressively in dating relationships, although at present we cannot be sure whether CPV is a precursor of dating violence or vice versa. In fact, Izaguirre and Calvete [33] in their longitudinal study found some evidence in favor of dating violence victimization as an antecedent of CPV. Taking into account the age of participants in this study, it is likely that CPV precedes dating violence, but there is no empirical evidence that supports this relationship. Children who were witnesses [57,58] or victims [58,59] of parental abuse will carry an increased risk of being violent into adulthood. Nevertheless, the literature shows that growing up in a violent family is not sufficient to engage in dating violence [39]. The results of this study indicate a mediational effect of CPV in the association between inter-parental violence exposure and dating violence, and a direct effect of inter-parental violence exposure on dating violence. Some studies have shown that the best predictor of violence in adulthood was the violence exercised against parents or siblings rather than that suffered as the intended recipient of such violence [40,57].

It was known that children exposed to inter-parental violence in childhood are more likely to show IPV as adults through the intergenerational transmission of violence hypothesis [22,57]. However, the effect size found in some studies was small [36,37]. Calvete et al. [37], in a longitudinal study, found that exposure to violence in a given year predicted an increase in dating violence in the following year. These authors indicated the mechanisms (disconnection and rejection schemas) through which inter-parental violence exposure and victimization of children lead to dating violence. In any case, this finding is explained by the social learning theory of Bandura [35], in which children exposed to inter-parental violence may witness positive consequences of aggression, which promotes the learning of aggression and the development of positive attitudes toward aggression [60] as a way of solving interpersonal conflicts. It seems that inter-parental violence exposure is not a sine qua non condition for persons who perpetrate violence toward their partner, because inter-parental violence had a small direct effect on dating violence. In dating relationships, the young person has the opportunity to repeat the maltreatment experience, as victim or as perpetrator of violence.

### 4.5. Mediational Effect of Sexism

The fifth hypothesis regarding the mediating effect of ambivalent sexism in the relationship between inter-parental violence exposure and dating violence was mostly exploratory because this is a new research area. A small indirect effect of inter-parental violence exposure through ambivalent sexism was found. According to the correlation matrix, father-to-mother violence exposure and ambivalent sexism were associated. This association could be explained by social learning theory. Children could internalize that mothers are intrinsically inferior to fathers through witnessing violent or disrespectful behavior by their father toward their mother. According to the correlation matrix, father-to-mother violence exposure and ambivalent sexism were associated. This association could be explained by social learning theory. Children could internalize that mothers are intrinsically inferior to fathers through witnessing violent or disrespectful behavior by their father toward their mother. It is necessary to replicate the study to test whether unidirectional father-to-mother violence is more related to ambivalent sexism than exposure to unidirectional mother-to-father or bidirectional violence. Moreover, it would be interesting to study in the future the intergenerational transmission of sexist beliefs.

Males showed more sexist beliefs than females, as in some previous studies in both college student populations [6,13] and community populations [12]. Ambivalent sexism explained 3% of dating violence while CPV explained 13%. Sexism is a set of negative beliefs relating to women that should be eradicated; it is one risk factor of dating violence among many others, with some studies based on university students showing low effect sizes [6,13,61]. A positive value of the present study is that it has focused on dating among heterosexual couples, given that it would not make sense to take negative beliefs about women into account if the partners are two members of the same sex. Sexual orientation is consolidated between the end of adolescence and the beginning of adulthood and should be taken into account when the relationship between sexism and dating violence is studied. Given the above analyses, it can be concluded that inter-parental violence exposure in college students is associated moderately with dating violence, with mediating effects of CPV and sexism.

### 4.6. Limitations

Previous research on CPV or inter-parental violence exposure typically focuses on children or adolescents. However, a sample based on adult children could be appropriate to study dating violence, CPV (although there are few studies), and even inter-parental violence exposure. Thus, a sample of adult children may indeed be an advantage. However, the main limitation of this study is that it is based on college students. As college students can come from more privileged families than from community population families, the number of individuals in our study witnessing physical parental violence in the past year was quite low, and results cannot be generalized to a community population. Another limitation to consider is the timing of the inter-parental violence variable because it was assessed only during the previous year of living together, being a study on inter-generational transmission of violence. It is possible that some participants were exposed to inter-parental violence earlier in childhood, but the objective of this study was to analyze the inter-parental violence exposure of early adulthood as a predictor of dating violence. These results may be generalized with caution to adolescent populations, taking into account that adult children will probably perpetrate less violence toward parents or toward their dating partner following Moffit trajectories [62].

Cross-sectional data makes it difficult to identify exactly how inter-parental violence exposure develops and influences other forms of violence (CPV and dating violence) over time. It is more likely that CPV occurs first and dating violence follows later. However, in this study, we cannot verify this, nor whether these two types of violence happen simultaneously. Although data provided by both partners would be preferable, in this study, only one of the partners reported data on both of them. As physical CPV (α ≥ 0.57) and dating violence (α ≥ 0.59) showed a level of internal consistency below the desirable level (α ≥ 0.70), results regarding prevalence rates should be interpreted with caution. The directionality of violence in dating relationships could not be analyzed because perpetration and victimization of violence were measured with different instruments. Since the chronicity of violence in interpersonal relationships is extremely important, it is recommended that it is recorded and analyzed in future studies. Another limitation of the present study is associated with the assessment of a dating violence model which involved the exclusion of those participants who had not had any dating relationships or were in same-sex dating relationships. It is worth noting that the dating violence model included participants who were in heterosexual dating relationships so as not to contaminate the results regarding ambivalent sexism, which may be differentially impacted in such situations. Unfortunately, however, we could not distinguish intimate partner violence among same-sex parents, which may affect inter-parental violence, although the proportion of same-sex parents would be lower than 2% [63].

## 5. Conclusions

In the relationship between exposure to inter-parental violence and intimate dating violence, child-to-parent violence and ambivalent sexism are intervening variables which facilitate dating violence. Social learning theory plays an important role in the intergenerational transmission of violence. Children learn models of interpersonal violence between parents by observation or by being abused or neglected by their parents. Dating violence is not a new issue in the scientific literature, but there is still much work to be done at both the theoretical and empirical levels. Researchers usually assess behaviors of physical, psychological, economic, or sexual violence of one partner toward the other in an intimate partner relationship because it is difficult to know the context in which IPV occurred. It would be interesting to assess whether women’s violence is a reaction to male violence, or if men initiate violence toward their partners and their partners then respond with violence, as well as who was injured [16]. Moreover, it would be important to recognize the inherent similarity in different types of interpersonal violence and family violence in order to advance in the understanding of dating violence. Nevertheless, the most important finding in the framework of the current study is that CPV by young adults could be an antecedent of dating violence perpetration. In these types of violence, it is recommendable to use a technical abuse approach instead of only zero tolerance.

Intervention models to prevent the perpetration of dating violence should include the prevention of inter-parental violence exposure during childhood and should pay special attention to young adults who have been exposed to this type of violence. The new knowledge about the role of CPV suggests the need for interventions to improve child–parent relationships while young people live with their parents, promoting respectful and prosocial behaviors between them. Interventions might need to assess the quality of the relationship between parents as well as the parent–child relationship, in order to improve relationships between family members and prevent dating violence. In addition, taking into account the rejection sensitivity model, intervention on the hostile perception of young perpetrators seems to be a key aspect to reduce both CPV and dating violence. Moreover, it is probable that perpetrators of violent behavior have traits of antisocial personality, depressive symptoms, or substance use [24,30,64].

Some erroneous beliefs about IPV or dating violence are changing, but greater effort is required to study other potential risk factors besides sexism. In general, levels of inter-parental violence, CPV, or dating violence could depend on cultural context as well as the relationships between these variables. For example, in a study conducted in Spain [25], inter-parental violence exposure was higher in immigrants than natives, while similar rates of CPV were found. In the same way, there were no differences with respect to IPV between immigrants and the native Spanish population [10]. In general, child and family services need to be sensitive to the cultural context of individuals involved in dating violence, and also to the gender of victims of this type of violence.

## Figures and Tables

**Table 1:** Prevalence rates, means, standard deviations, and correlations among observed variables in the model.

Variables	%/*M*	*SD*	1	2	3	4	5	6	7	8
*Inter-parental physical violence*										
1. Father-to-mother violence	6.2%/1.07	0.31	-							
2. Mother-to-father violence	6.4%/1.07	0.33	0.323**	-						
*Sexism*										
3. Hostile sexism	2.10	0.72	0.076*	0.071	-					
4. Benevolent sexism	2.06	0.67	0.105**	0.046	0.658**					
*Child-to-parent violence*										
5. Physical violence	24%/1.13	0.28	0.071*	0.237**	0.117**	0.096**	-			
6. Psychological aggression	94%/1.99	0.63	0.059	0.151**	0.001	0.005	0.367**	-		
*Dating violence*										
7. Perpetration	34%/1.09	0.18	0.189**	0.173**	0.110**	0.114**	0.199**	0.194**	-	
8. Victimization	74%/1.17	0.24	0.138**	0.095**	0.204**	0.181**	0.186**	0.169**	0.505**	-
*Demographic*										
9. Female	67%	-	0.078*	0.044	−0.265**	−0.220**	−0.098**	0.085*	0.108**	−0.008

* *p* < 0.05, ** *p* < 0.01
